# The Unintentional Procrastination Scale

**DOI:** 10.1007/s10942-016-0247-x

**Published:** 2016-08-11

**Authors:** Bruce A. Fernie, Zinnia Bharucha, Ana V. Nikčević, Marcantonio M. Spada

**Affiliations:** 1 Department of Psychology, Institute of Psychiatry, Psychology and Neuroscience, King’s College London, Henry Wellcome Building, De Crespigny Park, London, SE5 8AF UK; 2 HIV Liaison Service, South London and Maudsley NHS Foundation Trust, London, UK; 3 Kingston University, Kingston upon Thames, UK; 4 London South Bank University, London, UK

**Keywords:** Procrastination, Metacognition, Unintentional procrastination, Depression, Anxiety

## Abstract

Procrastination refers to the delay or postponement of a task or decision and is often conceptualised as a failure of self-regulation. Recent research has suggested that procrastination could be delineated into two domains: intentional and unintentional. In this two-study paper, we aimed to develop a measure of unintentional procrastination (named the Unintentional Procrastination Scale or the ‘UPS’) and test whether this would be a stronger marker of psychopathology than intentional and general procrastination. In Study 1, a community sample of 139 participants completed a questionnaire that consisted of several items pertaining to unintentional procrastination that had been derived from theory, previous research, and clinical experience. Responses were subjected to a principle components analysis and assessment of internal consistency. In Study 2, a community sample of 155 participants completed the newly developed scale, along with measures of general and intentional procrastination, metacognitions about procrastination, and negative affect. Data from the UPS were subjected to confirmatory factor analysis and revised accordingly. The UPS was then validated using correlation and regression analyses. The six-item UPS possesses construct and divergent validity and good internal consistency. The UPS appears to be a stronger marker of psychopathology than the pre-existing measures of procrastination used in this study. Results from the regression models suggest that both negative affect and metacognitions about procrastination differentiate between general, intentional, and unintentional procrastination. The UPS is brief, has good psychometric properties, and has strong associations with negative affect, suggesting it has value as a research and clinical tool.

## Introduction

Procrastination is a term familiar to many. It refers to the postponement or avoidance of starting, engaging in, and completing a task or a decision-making process. It is not a behaviour limited to students, despite high estimates of its prevalence in this population (up to 70 % according to Ellis and Knaus 1977). For example, the prevalence of procrastination has been found to be as high as 20 % in an adult sample (Harriott and Ferrari 1996). Within psychology, procrastination has been conceptualised as a failure of self-regulation (Baumeister and Heatherton 1996; Baumeister et al. 1994) associated with poor academic and work performance and mental ill health (Stöber and Joormann 2001).

The measurement of procrastination is vital to aid research that aims to understand this common and injurious behaviour. A range of self-report scales have been developed to measure this construct in general (e.g., the General Procrastination Scale or ‘GPS’: Lay 1986), in specific aspects (e.g., the Decisional Procrastination Scale or ‘DPS’: Mann 1982), and in specific populations (e.g., for students with the Tuckman Procrastination Scale or ‘TPS’: Tuckman 1991). More recently, the construct of intentional procrastination has been delineated and measured using the active procrastination scale (APS: Choi and Moran 2009). This separation of sub-types of procrastination is similar to that proposed by Ferrari (1993) who distinguished between functional and dysfunctional procrastinators, where the latter are characterised by their tendency to chronically delay starting or completing tasks.

The APS was developed to advance earlier work by Chu and Choi (2005) that proposed that there are two types of procrastinator: passive and active. A passive procrastinator was described as similar to more ‘traditional’ conceptualizations of the behaviour: i.e., the term refers to individuals who typically leave tasks to the last minute despite their good intentions, impairing performance. On the other hand, active procrastinators choose to delay task initiation or completion; they intend to procrastinate as a means of optimising performance. Indeed, Choi and Moran (2009) reported evidence that active procrastination was distinct from traditional procrastination, finding different relationships between measures of the former and latter with other study variables, such as participants’ self-reported performance and ability to structure their time (in a sample of Canadian university students). However, we argue that the items comprising the measure of passive procrastination used in their study did not clearly distinguish between intentional and unintentional procrastination, offering further justification of the need to develop a scale that entirely focuses on the latter. Indeed, a brief and informal content analysis of many of the extant procrastination scales appeared to offer support for the contention that several of their items fail to clearly distinguish between intentional and unintentional procrastination (e.g., the DPS, GPS, and TPS).

Intentional or active procrastination thus alludes to the idea that procrastination is to some extent a voluntary strategy, possibly aimed at self-regulation (Fernie and Spada 2008). If this were the case, active procrastination should correlate with positive beliefs about the benefits of engaging in procrastination, such as “Procrastination helps me cope” and “Procrastination allows to creativity occur more naturally” (Fernie and Spada 2008; Fernie et al. 2009). Research has also shown that individuals hold negative beliefs about procrastination, such as “When I procrastinate, I find it difficult to concentrate on other tasks” and “My procrastination is uncontrollable” (Fernie and Spada 2008; Fernie et al. 2009), concurrently with positive beliefs. This suggests that although procrastination may be voluntarily initiated for some, it later becomes, or is perceived as becoming, involuntary. These beliefs further support the conceptualisation of two domains of procrastination: intentional, or active, and unintentional.

Distinguishing unintentional from intentional procrastination may help to explain the strong relationship between worry, metacognitions pertaining to worry, and general procrastination (e.g., Spada et al. 2006; Stöber and Joormann 2001). Indeed, mindfulness, which can be defined as an awareness of the present (Brown and Ryan 2003) and thus, in some sense, representative of a meta-level of cognition, has been shown to mediate the relationship between procrastination and stress (Sirois and Tosti 2012). In addition, it is possible that procrastination may be appear to be uncontrollable because an individual’s cognitive resources are engaged in worry, leaving less mental assets available for task initiation or completion. If an individual is engaged in what they perceive as unintentional procrastination, they may attempt to regulate their predicament with further procrastination, leading to its perseveration.

Despite the myriad of pre-existing measures of procrastination, there is arguably a need for a brief, domain-specific measure of unintentional procrastination appropriate not just to students, but also to the general population. We hypothesized that unintentional procrastination is likely to be a stronger marker of psychopathology than general procrastination because it encapsulates perceived involuntary and not voluntary control of this behaviour, thus a measure of unintentional procrastination might not only be beneficial for research, but also for use in clinical settings. The current paper reports two studies that trace the development of the Unintentional Procrastination Scale. The first study incorporates a principle components analysis and an assessment of internal consistency to construct the scale. The second details a confirmatory factor analysis using data from a second sample as well as assessment of the construct and divergent validity of the newly constructed scale, specifically considering the likelihood of shared proportion of variance explained by general and unintentional procrastination. We also explored the relationships between negative affect with intentional, unintentional, and general procrastination.

## Study 1: Construction of the Unintentional Procrastination Scale

### Method

#### Participants

A convenience sample of 139 (111 female; mean age = 29.5 years [SD = 11.5; range 18–72 years]) participants was recruited for this study and completed a battery of online questionnaires. Eligibility criteria were minimal to attract a sample that represented a broad range of individuals. Participants were required: (1) to be at least 18 years of age, (2) to possess adequate English language skills, and (3) to consent to participate.

The sample was international, with participants reporting nationalities from six continents, although the distribution of participants’ nationalities was skewed, with 46.8 % (65) reporting to be British. The ethnicity of the sample was also skewed, with 67.6 % (94) of participants reporting to be white. Because an international sample was anticipated, it was thought likely that for many of the participants English would not be their first language. Indeed, 38.8 % (54) of the sample reported a first language other than English. As a result, participants were asked to rate their confidence in their ability to speak, read, and write in English. 96.4 % (133) of the sample rated their confidence in all three of these domains as either ‘confident’ or ‘very confident’.

From earlier research that has developed questionnaires, a frequently identified limitation is a failure to control for participants’ previous (or current) exposure to psychological therapies and how this may impact on responses to items in questionnaires (e.g. Fernie et al. 2014). Thirty-four participants reported that they had undergone psychotherapy previously that is not on going. Of these, 58.8 % (20) stated that CBT had been a component in their therapy, 70.6 % (24) had seven or more sessions, and 14.7 % (five) reported that at least part of their therapy aimed to address problematic procrastination. 6.5 % (nine) participants reported that they were currently in psychotherapy, with 22.2 % (two) of these stating that they were being treated with CBT, 44.4 % (four) declaring that they had completed more than seven sessions, and 66.7 % (six) revealing that problematic procrastination was being addressed in their therapy.

In an attempt to control for variations in mental health wellbeing, self-report mood measures were taken and participants were also asked if they have a psychiatric diagnosis from a mental health clinician. 9.4 % (13) of participants reported that they had, with the majority reporting diagnoses of depression and anxiety disorders.

#### Materials

Seven items pertaining to unintentional procrastination were derived from a review of transcriptions of an earlier procrastination study (Fernie and Spada 2008), as well as the authors’ clinical experience and from deductions based on theory to form the raw version of the Unintentional Procrastination Scale (UPS).

Items were framed as statements to which participants could respond to on a four-point Likert-type scale to indicate their level of agreement (“1. Do not agree”, “2. Agree slightly”, “3. Agree moderately”, and “4. Agree strongly”). The items were preceded by a pre-amble that read as follows:

Please read each statement and select a number 1, 2, 3 or 4 that indicates how much you agree or disagree with the statement. There are no right or wrong answers. Do not spend too much time on any statement.

#### Procedure

Participants were recruited via social media and through a London university’s fortnightly research volunteer email circular. An additional recruitment strategy involved emailing a hyperlink to the online questionnaires to individuals on the authors’ email contact lists and asking recipients to forward this on to others on their contact lists, in attempt to create a viral-like spread.

Potential participants were directed to the study website containing the questionnaires. The first two pages of this provided information regarding the purpose of the study, how responses were anonymous, and that consent would be assumed once participants click on the ‘submit’ button that followed the battery of questionnaires. The pages following this information contained a series of questions to ascertain participants’ demographic details, their exposure to psychotherapy, and their current mental wellbeing (i.e. do they have a psychiatric diagnosis?). Participants were not required to record their names.

### Results

#### Principle Components Analysis

The seven original items of the UPS were subject to a principle components analysis (PCA). Both initial eigenvalues (only a single component had a value over one) and a Scree plot indicated a one-factor solution. This single factor accounted for 60.7 % of the variance and all seven items loaded strongly (see Table 1). The internal consistency of the newly developed scale was .89 and further analysis indicated that this would not be improved if any of the items were removed.

**Table 1:** Factor Loadings for the UPS items from Initial PCA, Second PCA, and Second CFA

	PCA loading (total sample; n = 139)	PCA loading (no CBT exposure; n = 105)	2nd CFA loadings (total sample; n = 131)
1. I rarely begin tasks as soon as I am given them, even if I intend to.	.643	.627	1.000
2. I find it difficult to make a decision the moment I am faced with it.	.411	.392	N/A
3. Often I mean to be doing something, but it seems that sometimes I just don’t get round to it.	.760	.749	1.179
4. I often seem to start things and don’t seem to finish them off.	.605	.590	1.269
5. I intend to get things done, but sometimes this just does not happen.	.634	.616	1.449
6. Often I will set myself a date by which I intend to get something done or make a decision, but miss the deadline.	.577	.577	1.429
7. I really want to get things finished in time, but I rarely do.	.620	.607	1.553

We re-ran the PCA excluding the participants that reported having current or previous CBT to control for exposure to procrastination-related cognitions. This led to the loading of item #2 to fall below .4 to .39. However, because a subsequent analysis with the ‘no current or previous exposure to CBT’ sample suggested that the internal consistency would not be improved by removing any of the items, we retained the original seven-item UPS for the second study where the factor would be subjected to a confirmatory factor analysis (CFA) using a new dataset.

## Study 2: Validation of UPS

### Introduction

In order to validate the UPS and provide evidence to support the delineation of procrastination into intentional and unintentional domains, we tested several hypotheses. Firstly, we hypothesized that UPS scores would be significantly correlated with those from a pre-existing measure of procrastination, establishing concurrent validity, and that this relationship would remain significant when controlling for negative affect, suggesting divergent validity. Secondly, we posited that as a test of divergent validity, because we have argued that unintentional procrastination is a stronger marker of psychopathology than both general and intentional procrastination, data from the UPS would be a stronger predictor of negative affect than measures of general and intentional procrastination. Thirdly, we predicted that the UPS would be more strongly associated with negative beliefs about procrastination than measures of general and intentional procrastination. Finally, we hypothesized that intentional procrastination would be a stronger predictor of positive beliefs about procrastination than UPS scores and a measure of general procrastination.

### Method

#### Participants and Procedure

A convenience sample of a 155 participants (118 female; mean age = 32.5 years [SD = 11.4; range 18–68 years]) completed a battery of online questionnaires. Eligibility criteria and the procedure matched that used in Study 1.

Again, the participants were international and the distribution of participants’ nationalities was skewed, with 46.2 % (72) reporting as British. The majority of the sample self-reported their ethnicity as white (71.6 % [110]) and 61.9 % (96) stating that English as their first language. At least 90.3 % (139) of participants rated their confidence in speaking, reading, or writing in English as either ‘confident’ or ‘very confident’.

In terms of exposure to psychological therapy, 1.9 % (3) of participants were currently in therapy and 24.5 % (38) had undergone it in the past. Overall, 7.4 % (12) of participants reported that they were, or had undergone, CBT. Only 8.4 % (13) of participants stated that they had a mental health diagnosis, reporting depressive, anxiety, or eating disorders.

#### Materials

##### Emotional Measures

The Patient Health Questionnaire 9 (PHQ-9; Kroenke et al. 2001) was used to assess depressive symptoms. The PHQ-9 is a nine-item scale that possesses good psychometric properties, with higher scores indicating the presence of greater levels of symptoms (Kroenke et al. 2001). The Generalized Anxiety Disorder 7 (GAD-7; Spitzer et al. 2006) was administered to measure anxiety symptoms. The GAD-7 also possesses good psychometric properties and higher scores again indicate the presence of more anxiety symptoms (Spitzer et al. 2006).

##### Procrastination Measures

In addition to the newly developed UPS, the General Procrastination Scale (GPS: Lay 1986) was used to assess traditional procrastination. The GPS is a 17-item questionnaire that taps largely into, arguably, both intentional and unintentional procrastination. The Active Procrastination Scale was also used and consists of a total of 16-items equally distributed over four factors, namely ‘preference for pressure’, ‘intentional decision to procrastinate’, ‘ability to meet deadlines’, and ‘outcome satisfaction’ (Choi and Moran 2009). This current study focuses on the ‘intentional decision to procrastinate’ (IDP) factor that consists of items such as “I intentionally put off work to maximize my motivation” and “To use my time more efficiently, I deliberately postpone some tasks”.

Finally, we used the Metacognitive beliefs about Procrastination scale (MaP) to assess higher-order thinking about procrastination (Fernie et al. 2009). The MaP consists of 16-items equally distributed over two subscales: positive metacognitions about procrastination (PMP) and negative metacognitions about procrastination (NMP). An example item of the PMP is “When I procrastinate, I am unconsciously mulling over difficult decisions” and for NMP is “My procrastination is uncontrollable”. NMP has been shown to significantly correlate with both general and decisional procrastination and PMP to only the latter (Fernie et al. 2009). The factors have been shown to possess good internal consistency (Fernie et al. 2009).

### Results

#### Confirmatory Factor Analysis

Responses to the seven-items from the UPS were used to confirm its single factor structure. The Lavaan package (Rosseel 2012) was installed into R Studio (R-Studio 2015) and was used to conduct a confirmatory factor analysis (CFA). We defined unintentional procrastination as the single latent variable and the seven-items of the UPS as congeneric indicators of the latent variable. Using maximum likelihood estimation, we assumed multivariate normality of item scores and defined them as continuous indicators within the model. We utilised four indices to evaluate the fit of the model: a Chi square measure of fit, the Root Mean Square Error of Approximation (RMSEA), the Comparative Fit Index (CFI), and the Tucker-Lewis Index (TFI: also known as the Non-Normed Fit Index).

This initial CFA revealed mixed results regarding the fitting of the data to the specified model. Two absolute fit indices suggested that the data weakly fitted the specified model: the Chi square test was significant (**χ** ^2^ = 29.13, *df* = 14, *p* > .01) and the RMSEA was 0.09. However, relative fit indices produced an opposite picture. The CFA generated a CFI of 0.97 and TLI of 0.95 suggesting that the data better fit the specified model than the baseline model. Parameter estimates were reviewed and modification indices were calculated. Together, these suggested that a re-specified model, resulting from the removal of a single item (#2), might lead to an improvement of fit. The re-specified model was a better fit of the data, with a non-significant Chi square test (**χ** ^2^ = 10.72, *df* = 9, *p* = .30), an RMSEA of 0.038, CFI of 0.99, and a TFI of 0.99. See Table 1 for the factor loadings for the re-specified model (note that Lavaan automatically assigns a loading of 1.000 to the first item).

#### Construct Validity

Table 2 shows the means, standard deviations, and ranges for all experimental variables. A series of Kolmogorov–Smirnov tests of normality were conducted on the data that suggested PHQ-9, GAD-7, PMP, NMP, and UPS were significantly different than normal, while the GPS and IDP were not. As a result a series of non-parametric, Spearman’s Rho correlation analyses were conducted on the data (see Table 2). These revealed that the UPS was positively associated with GPS (very strong), PMP (moderate), NMP (moderate), IDP (weak), PHQ-9 (strong), and GAD-7 (strong).

**Table 2:** Means, SDs, and ranges for all experimental variables and correlation matrix

	Means	SD	Range	1	2	3	4	5	6	7
1. UPS	13.67	4.91	6 to 24		.78^**^	.31^**^	.35^**^	.25^**^	.53^**^	.47^**^
2. GPS	−0.04	14.31	−28 to 37			.26^**^	.23^**^	.22^*^	.47^**^	.49^**^
3. PMP	14.99	4.57	8 to 32				−.07	.34^**^	.28^**^	.25^**^
4. NMP	18.83	6.97	8 to 32					.02	.45^**^	.50^**^
5. IDP	15.97	5.11	4 to 28						.25^**^	.19^*^
6. PHQ-9	14.96	5.9	9 to 35							.73^**^
7. GAD-7	12.36	5.45	7 to 28							

*UPS* Unintentional Procrastination Scale, *GPS* General Procrastination Scale, *PMP* Positive Metacognitions about Procrastination, *NMP* Negative Metacognitions about Procrastination, *IDP* Intentional Decision to Procrastinate, *PHQ*-*9* Patient Health Questionnaire, *GAD*-*7* General Anxiety Disorder-7, *n* = 118 to 131; * *p* < .05; ** *p* < .01

In order to further test the construct validity of the UPS, we assessed its relationship with GPS while controlling for IDP and negative affect. The relationship between UPS and GPS remained significant (**β** = 0.79, *p* < .001 [LL = 1.96, UL = 2.09]). In this model, which accounted for 67 % of the variance in the pre-existing measure of procrastination, IDP, GAD-7, and PHQ-9 became non-significant predictors of GPS.

#### Unintentional Procrastination as a Marker of Psychopathology

We conducted two further regression analyses, the first with GAD-7 as the dependent variable and the second with PHQ-9, to test the hypothesis that unintentional procrastination would be a stronger marker of psychopathology than measures of intentional and general procrastination. The same sets of predictor variables were entered into both models: UPS, IDP, and GPS. In both models, only UPS remained a significant predictor: both with PHQ-9 as the dependent variable (**β** = 0.52, *p* < .001 [LL = 0.31, UL = 0.94]) and GAD-7 (**β** = 0.48, *p* = .001 [LL = 0.23, UL = 0.83]). Thus, despite the shared pattern of correlations between the measures of negative affect and the IDP, GPS, and UPS, these analyses provided some evidence of divergent validity existing between all three measures.

#### Metacognitions and Intentional and Unintentional Procrastination

We predicted that unintentional procrastination would be more strongly associated with negative metacognitions about procrastination than measures of intentional and general procrastination. We tested this by conducting a regression analysis with NMP as the dependent variable and UPS, IDP, and GPS as independent variables. In this model, UPS once more was the only significant predictor of NMP (**β** = 0.52, *p* = .001 [LL = 0.33, UL = 1.17]).

To test our hypothesis that intentional procrastination would be a stronger predictor of positive metacognitions about procrastination than both general and unintentional procrastination, we calculated another regression analysis with PMP as the dependent variable and UPS, IDP, and GPS as independent variables. In line with our hypothesis, only IDP was a significant predictor of PMP (**β** = 0.27, *p* < .001 [LL = 0.09, UL = 0.40]).

Finally, to see if participants’ exposure to psychotherapy impacted on their scores on the measures of procrastination used in this study, a series of Mann–Whitney U or independent *t* tests were used (dependent on the distribution of data) to compare these two groups. None of these tests resulted in significant results: i.e., GPS [t(116) = 1.73, *p* = .08; n_exposure_ = 32, n_no-exposure_ = 86], IDP [t(107) = 0.42, *p* = .68; n_exposure_ = 30, n_no-exposure_ = 79], PMP [U = 1072, *p* = .39; n_exposure_ = 30, n_no-exposure_ = 80], and NMP [U = 1191, *p* = .95; n_exposure_ = 30, n_no-exposure_ = 80].

#### Assumptions of Regression Analyses

A total of five regression analyses were conducted for this study and the suitability of the data for all the models for this kind of analyses was assessed. Firstly, there was no evidence of multicollinearity in the dataset for all models: (1) no correlations greater than *r* = .9 were identified between the predictor variables used in the regression analyses (the strongest correlation was found between GPS and UPS at *r* = .78); (2) the Tolerance Index (TI) values were all above .20 (e.g., for all of the models, the predictors’ TIs ranged between .27 and .93); (3) the Variance Inflation Factors (VIFs) for all predictor variables were substantially less than 10 (e.g., again, for all of the models, the predictors’ VIFs ranged between 1.08 and 3.78); and (4) eigenvalues, condition indexes, and variance proportions were calculated for all models. Models that used both GPS and UPS as predictors revealed that both of these variables explained large variance proportions (>50 %) at the smallest eigenvalues, however none of these were associated with condition indexes greater than 15. This provided further evidence that, despite the strong correlation between GPS and UPS, as well as their shared patterns of correlations between GAD-7 and PHQ-9, multicollinearity was not problematic for any of the models. Secondly, the Durbin-Watson test suggested that the assumption of independent errors is tenable. Thirdly, histograms and normality plots suggested that the residuals were normally distributed and plots of the regression-standardized residuals against the regression standardized predicted values suggested that the assumptions of linearity and homoscedasticity were met.

## Discussion

The central aim of this study was to develop a brief measure of unintentional procrastination in the form of the UPS. This study resulted in a six-item measure of unintentional procrastination that appeared to possess construct and divergent validity and good internal consistency. The final six-item version of the UPS was a good fit of Study 2 data. The UPS remained a strong predictor of general procrastination even when controlling for negative affect.

Our second set of hypotheses regarded unintentional procrastination being a stronger marker of psychopathology than both general and intentional procrastination. Despite the strong correlation between the GPS and the UPS (acknowledging that general and unintentional procrastination are overlapping concepts), our tests of multicollinearity and our regression models supported this hypothesis, as UPS scores were independent predictors of both anxiety and depression in models that contained measures of general and intentional procrastination.

**Fig. 1:**
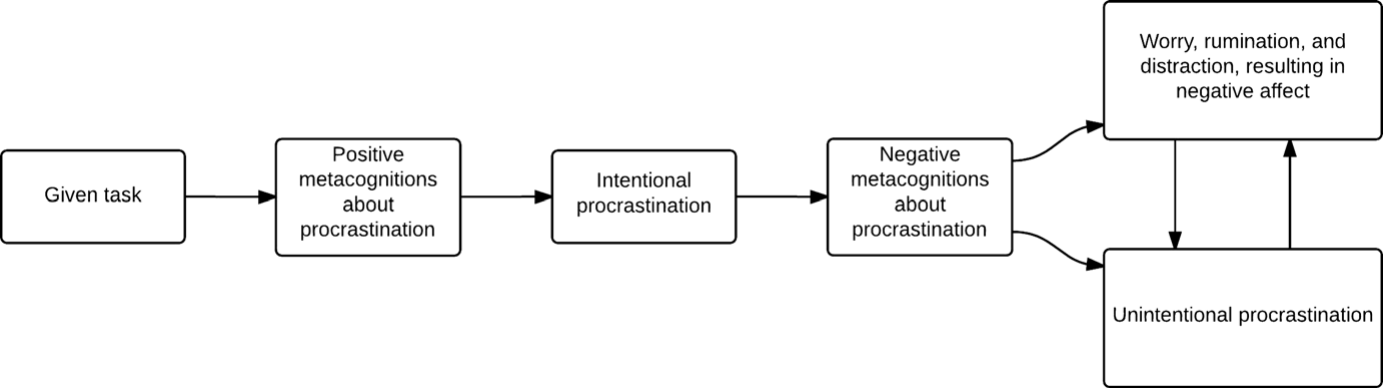
Speculative metacognitive model of procrastination

Our final set of hypotheses concerned the role of metacognitions about procrastination in the delineation between intentional and unintentional procrastination. Our results suggested that positive metacognitions about procrastination are more strongly associated with intentional procrastination, while negative metacognitions are more relevant to unintentional procrastination. It is possible to speculate a metacognitive model of procrastination to suggest how these relationships may operate. Firstly, an individual with positive beliefs about procrastination might respond to being given a task with intentional procrastination. Examples of these metacognitions might include “Procrastination allows creativity to occur more naturally” and “Procrastination stops me from being bored” (Fernie et al. 2009), therefore relating to not only to optimising performance, but also to minimising feelings of discomfort. Secondly, intentional procrastination, in problematic or dysfunctional procrastinators (Ferrari 1993), could activate negative beliefs about procrastination, leading to negative affect. Unintentional procrastination might be responded to with cognitive processes that are ‘resource-heavy’ (such as worry, rumination, and distraction) in a maladaptive attempt to control this behaviour. Such responses might be activated because an individual holds certain other positive metacognitions pertaining to these processes. For example, an individual might respond with worry because they believe it helps them to organize their thoughts (Cartwright-Hatton and Wells 1997) or with rumination because of metacognitions such as “Ruminating about the past helps me to work out how things could have been done better” (Papageorgiou and Wells 2001). Responses such as these could result in a depletion of mental resources that inhibit an individual from achieving their optimal performance, reinforcing negative self-efficacy beliefs and maintaining the postponement of starting or completion of a task or the making of a decision (see Fig. 1), resulting in what Ferrari (1993) referred to as dysfunctional procrastination.

This study is subject to several limitations that will have to be addressed by future research. First, social desirability, self-report biases, context effects, and poor recall may have contributed to errors in the self-report measurements. Future studies could involve Ecological Momentary Assessment to test whether UPS scores predict real-time procrastination, further establishing construct validity. Second, a cross-sectional design was adopted and this does not allow causal inferences. Third, this study utilized self-report measures to assess subjective experience and meta-awareness and, as such, like much cognitive research, there is always doubt whether we are measuring the constructs we intend. Fourth, there were issues with the sample characteristics: it was moderate in size and this impacted on the power of the statistical analyses; the majority of participants were female; and participants predominately ethnically identified themselves as ‘white’ and in terms of nationality, British. This impacts on our ability to generalize these findings to other ethnicities and nationalities, though a significant proportion of participants self-reported as non-white and non-British. Fifth, the lack of homogenous sample nationality risked leading to increased error measurements due to the self-report measures all being written in English; however, participants’ ratings of their language abilities suggested that very few were not confident in English. Finally, the strong correlation between GPS and UPS data raises concerns that they are measuring similar constructs. However, we assumed that general procrastination would encapsulate both intentional and unintentional aspects (e.g., the GPS was significantly correlated with both IDP and UPS). Furthermore, our collinearity diagnostics that we employed for our regression models provided evidence that the GPS and the UPS measure similar but distinct constructs.

Despite these limitations, we believe that the UPS is promising research tool. It is brief and easy to administer; its use is not limited to specific samples (e.g., students) and it strongly correlates with pre-existing measures of procrastination. Perhaps the most important contribution this scale makes is by providing further evidence for the delineation of procrastination into intentional and unintentional domains, and by suggesting that the latter is stronger marker of psychopathology than the former.

## Unintentional Procrastination Scale

Please read each statement and select a number 1, 2, 3 or 4 that indicates how much you agree or disagree with the statement. There are no right or wrong answers. Do not spend too much time on any statement.

**Table:** 

	Do not agree	Agree slightly	Agree moderately	Agree very much
1. I rarely begin tasks as soon as I am given them, even if I intend to.	1	2	3	4
2. Often I mean to be doing something, but it seems that sometimes I just don’t get round to it.	1	2	3	4
3. I often seem to start things and don’t seem to finish them off.	1	2	3	4
4. I intend to get things done, but sometimes this just does not happen.	1	2	3	4
5. Often I will set myself a date by which I intend to get something done or make a decision, but miss the deadline.	1	2	3	4
6. I really want to get things finished in time, but I rarely do.	1	2	3	4
